# A Case of Urethral Duplication Arising from the Posterior Urethra to the Scrotum with Urinary Stone in a 6-Year-Old Male

**DOI:** 10.1155/2014/290623

**Published:** 2014-11-25

**Authors:** Kenichi Mori, Toshitaka Shin, Shohei Tobu, Mitsuru Noguchi, Yasuhiro Sumino, Fuminoi Sato, Hiromitsu Mimata

**Affiliations:** ^1^Department of Urology, Oita University Faculty of Medicine, Idaigaoka 1-1, Hasama-cho, Yufu, Oita Prefecture 879-5593, Japan; ^2^Department of Urology, Saga University Faculty of Medicine, Nabeshima 5-1-1, Saga, Saga Prefecture 849-8501, Japan

## Abstract

Urethral duplication is a rare congenital anomaly. We report a 6-year-old male with type IIA2 (Y-type) using Effmann's classification. The accessory urethra, in which a urinary stone existed, arose from the posterior urethra to the scrotum. Because of recurrent urinary tract infection and urinary discharge from the accessory urethra, surgical removal of the accessory urethra through a scrotal incision was performed. At 7-month postoperative follow-up the patient was completely free from urinary incontinence and urinary tract infection.

## 1. Introduction

Patients with urethral duplication, an extremely rare congenital anomaly, can present with incontinence, urinary tract infection (UTI), and double stream or can be asymptomatic with the duplication being found on routine physical examination [1]. We report a 6-year-old male with scrotal sinus as a variant of urethral duplication arising from the posterior urethra to the scrotum.

## 2. Case Report

**Figure 1:**
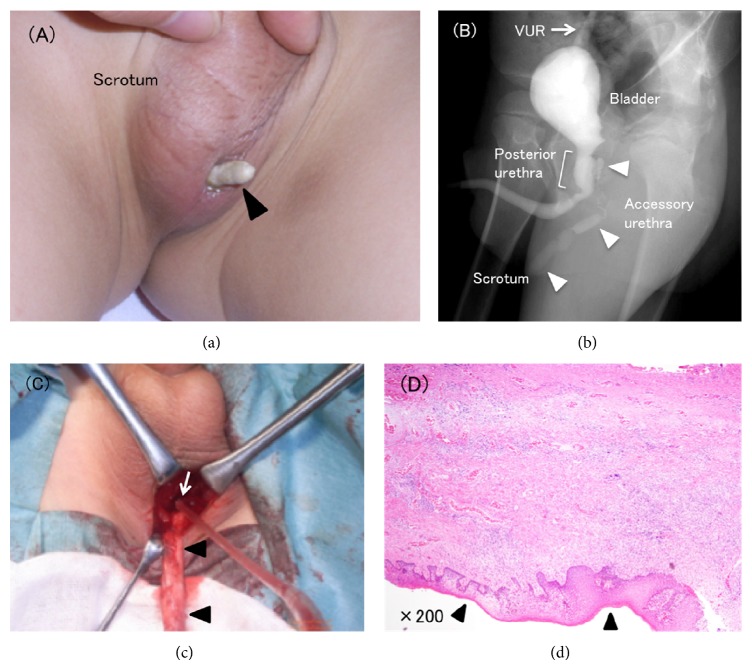
(a) The opening of the sinus with urinary stone (arrowhead) in the midline scrotum. (b) VCUG demonstrated the accessory urethra arising from the posterior urethra to the scrotum (arrowheads) and right grade I VUR (arrow). (c) The 4.5 cm long accessory urethra (arrowheads) was removed through a scrotal incision. White arrow was the junction of posterior urethra and accessory urethra. (d) The sinus lumen was lined by squamous epithelium with keratinization (arrowheads). Capillary vessels and smooth muscle tissue were observed in subepithelium (HE, ×200).

A 6-year-old boy with a history of pale yellow discharge from the opening of the sinus and a stony excrescence of the sinus in the midline scrotum was evaluated (Figure 1(a)). Other physical findings were normal. However, urine analysis showed slight pyuria. Voiding cystourethrogram (VCUG) demonstrated communication between the sinus and the posterior urethra and right grade I vesicoureteral reflux (VUR) (Figure 1(b)). Computed tomography and magnetic resonance imaging also revealed communication between the sinus and posterior urethra. As a result of examination, urethral duplication arising from the posterior urethra to the scrotum was mostly suspected as the clinical diagnosis.

Cystourethroscopy, retrograde urethrography (RUG), sinus graphy, and surgical removal of the sinus through a scrotal incision were performed simultaneously. The patient was placed in lithotomy position. An elliptic incision was accomplished in the skin around the opening and the surrounding tissues were delicately dissected up to near the bifurcation of the urethra. The ectopic channel received double ligation with 3-0 polyglactin, and it was excised. The subcutaneous tissue was closed with 4-0 polyglactin 910 and the skin was sutured with 4-0 polydioxanone. The 4.5 cm long sinus extended to the left side of the verumontanum (Figure 1(c)). No posterior urethral membrane was involved in the urethral obstruction. Histologically, the sinus lumen was lined by squamous epithelium and surrounded by smooth muscle and capillary vessels (Figure 1(d)). Based on this result, the sinus was finally diagnosed as an accessory urethra of urethral duplication [2].

## 3. Discussion

Urethral duplication is a rare anomaly with about 300 cases reported to date, usually seen in males and often associated with genitourinary and gastrointestinal anomalies [3]. Urethral duplication can be classified into three types using Effmann's classification [4]. One-third of patients have associated VUR. In type I, the most common type, partial duplication of the urethra is observed and is almost always asymptomatic, requiring no treatment. In type II, complete duplication of the urethra is observed. Type II urethral duplication can be classified as type IIA1 if both urethras arise from the separate bladder necks, type IIA2 (Y-type duplication) if one channel arises from the other, and type IIB if duplication with one meatus is observed. Type III urethral duplication comprises complete duplication of the urethra and bladder. Embryonically, urethral duplication is not well understood and various hypotheses exist. Urethral duplication can be caused by the growth arrest of the urogenital sinus [5] or abnormal Müllerian duct termination or misalignment of the termination of the cloacal membrane with genital tubercle [6]. Depending on duplication type, patients may be asymptomatic. Symptoms include UTI, epididymitis, and incontinence [7]. Diagnosis can be made using VCUG or RUG. Urodynamic study helps to confirm the position of the functional urethra to distinguish it from congenital urethroperineal fistula.

Our case was classified as type IIA2 (Y-type) because of the accessory urethra arising from the posterior urethra to the scrotum. In almost all cases of Y-type, the accessory urethras were opened on the perineum or rectum arising from orthotropic urethra. The primary aim of surgical repair of Y-type urethral duplication is to preserve normal functioning orthotopic urethra with intact verumontanum, good caliber, and intact sphincter [8]. Surgical management should be planned individually according to the anatomical findings of the abnormality. In this case, because of recurrent UTI and urinary discharge from the accessory urethra, surgical removal of the accessory urethra through a scrotal incision was performed. At 7-month postoperative follow-up the patient was completely free from urinary incontinence, UTI, and VUR.

In conclusion, urethral duplication is a rare congenital anomaly, either isolated or associated with other anomalies, with varied presentations and requires radiologic and also endoscopic examination to define the anatomy and planning of the surgical approach.
